# Discrepancy between objective and subjective cognition in adults with major depressive disorder

**DOI:** 10.1038/s41598-017-04353-w

**Published:** 2017-06-20

**Authors:** Manit Srisurapanont, Sirijit Suttajit, Kanokkwan Eurviriyanukul, Prirada Varnado

**Affiliations:** Department of Psychiatry, Faculty of Medicine, Chiang Mai University, Chiang Mai, 50200 Thailand

## Abstract

This study aimed to determine: i) the correlation between objective and subjective cognition, ii) the correlates of objective and subjective cognition and iii) the predictors of discrepancy between objective and subjective cognition. Participants were non-elderly patients with major depressive disorder (MDD). We assessed subjective cognition using the Perceived Deficit Questionnaire for Depression (PDQ-D) and objective cognition using Face I and Face II tests of the Wechsler Memory Scale, 3rd edition and Digit Span and Matrix Reasoning tests of the Wechsler Intelligence Scale for Adults, 3rd edition. The discrepancy between objective and subjective cognition was estimated. Participants were 57 outpatients with MDD. PDQ-D scores were not correlated with composite neurocognitive test (NCT) *z* scores. Years of education significantly predicted composite NCT *z* scores, as did age. The 9-item Patient Health Questionnaire (PHQ-9) scores significantly predicted PDQ-D scores, as did antidepressant treatment. Age significantly predicted discrepancy scores, as did PHQ-9 scores. In conclusion, objective and subjective cognition in patients with MDD are not correlated. Age and education predict objective cognition. Depression. severity and antidepressant treatment predict subjective cognition. Age and depression severity may predict the discrepancy between objective and subjective cognition.

## Introduction

Cognition is a generic term embracing the mental activities associated with thinking, learning, and memory^1^. Cognitive dysfunction is common in depressed patients. While ‘hot’ cognitive dysfunction implies a cognitive bias toward negative information and a misinterpretation of social cues, ‘cold’ cognitive dysfunction is the functional impairment of information process in the absence of any emotional influence^2^. Hot cognitive dysfunction is highly correlated with depression and may contribute to the perpetuation of negative emotional states in MDD^3^. Depressed patients perform neurocognitive or cold cognitive tests poorer than healthy controls in many areas^4^. In addition, they also have more cognitive complaints than non-depressed patients^5^. Several lines of evidence have shown that neurocognitive and perceived cognitive dysfunction in depressed patients is associated with functional disability and adverse outcomes^6–8^.

Not only objective but also subjective cognition is of concern for depressed patients. Up to 81% of patients with MDD may have subjective cognitive dysfunction, e.g., memory or concentration complaints^8^. As a diagnostic criterion for major depressive disorder, not only objectively but also subjectively diminished ability to think, as well as concentrate, or indecisiveness can be observed^9^. Recent evidence suggests that subjective cognitive dysfunction more closely relates with functioning than an objective dysfunction^10^.

Subjective cognition in depressed patients remains unclear. Growing evidence has raised questions regarding the correlation between objective and subjective cognition^11, 12^. While an earlier study did not find the correlation between subjective cognitive dysfunction and functional disability^13^, two recent studies found such an association^11, 14^.

Studies on the predictors of objective and subjective cognition, as well as their discrepancy, would be helpful to clarify the similarity and/or distinction between objective and subjective cognition in depressed patients. In this study, we proposed to: i) determine the correlation between objective and subjective cognition, ii) examine the socio-demographic and clinical correlates of objective or subjective cognition and iii) identify the predictors of discrepancy between objective and subjective cognition.

## Methods

Participants in this study were a subgroup of those taking part in ‘The Study of Cognitive Dysfunction in Asians with Depression’ or CogDAD study^15^. In addition to the assessment of subjective cognition in the main project, we added a number of neurocognitive tests for assessing objective cognition at our study site. Both the CogDAD and the add-on studies were cross-sectional, observational studies concurrently carried out at the Psychiatric Clinic, Maharaj Nakorn Chiang Mai Hospital, a university hospital providing tertiary care in Northern Thailand. All assessments were completed in a single visit. Both study protocols were approved by the Ethics Committee of Faculty of Medicine, Chiang Mai University. All the participants provided written informed consent prior to participation in the studies. All methods used in the study were performed in accordance with the guidelines given and the regulations overseen by the Ethics Committee.

### Participants

Our study enrolled outpatients who met the DSM-IV criteria for major depressive disorder between 21 and 65 years old. Exclusion criteria were no history of schizophrenia or psychotic disorders, bipolar disorder, dementia or any other neurodegenerative disease, alcohol/substance dependence, substance-induced mental disorders or psychiatric disorders due to a general medical condition/substances.

### Assessment

We used the Perceived Deficits Questionnaire for Depression (PDQ-D) to assess subjective cognition^16^. This questionnaire was modified from the PDQ developed for assessing cognitive dysfunction in patients with multiple sclerosis^17^. The PDQ-D has high internal consistency with a Cronbach’s alpha of 0.81 and strongly correlated with other measures of cognitive functioning. In addition, it is moderately correlated with several construct measures known to be associated with cognitive functioning^18^. Each of its 20 items asked about the frequency of an experience of cognitive deficit in the past week, which was rated as 0 (never), 1 (rarely), 2 (sometimes), 3 (often) and 4 (almost always). The questionnaire consisted of four subscales, including Attention/Concentration, Retrospective Memory, Prospective Memory and Planning/Organization. PDQ-D scores range from 0–80; and the higher PDQ-D scores, the poorer the subjective cognition.

To match with the components of PDQ-D, we used four neurocognitive tests for assessing objective cognition. Two tests for memory performance included Face I and Face II tests of the Wechsler Memory Scale, 3rd edition (WMS-III)^19^. The other two tests for attention and executive function were Digit Span and Matrix Reasoning tests of the Wechsler Intelligence Scale for Adults, 3rd edition.(WAIS-III)^20^, respectively. The higher the score of each test, the better objective cognition in that area.

Patients rated their depression severity by using the 9-item Patient Health Questionnaire (PHQ-9), the official Thai version^21^. PHQ-9 scores range from 0 to 30. The higher the PHQ-9 scores, the more severe the depression. We used the Clinical Global Impression, Severity scale to measure the overall severity of mental illness^22^.

Patients rated their disability by using the Sheehan Disability Scale (SDS)^23^. This three-subscale measure was developed to assess perceived disability in three areas, including work/school, social life/leisure and family/home life. The scores of each area ranged from 0 (not at all) to 10 (extremely). A SDS subscale score of five or more on any of the three areas was defined as a significant disability^24^.

Complete standard linguistic validation processes, including forward-translation, backward-translation and cognitive briefing, were applied to develop the Thai versions of PDQ-D and SDS.

### Statistical Analyses

Both the units and score ranges of Face I, Face II, Digit Span and Matrix Reasoning tests were dissimilar. We, therefore, transformed the raw scores of these neurocognitive tests into standardized (*z*) scores and combined them as unweighted composite *z* scores comprising four neurocognitive tests, called composite NCT *z* scores. This composite NCT *z* score was considered as a measure of overall objective cognition.

To make PDQ-D total scores comparable to composite NCT *z* scores, we transformed raw PDQ-D scores into *z* scores. However, compared to composite NCT *z* scores, PDQ-D *z* scores were still interpreted in the opposite direction. While higher composite NCT *z* scores suggest higher objective cognition, higher PDQ-D *z* scores indicate poorer subjective cognition. To make the *z* scores of both measures comparable, we reversed the values of PDQ-D *z* scores (eg, +1 to −1, −1 to +1). Composite NTC and PDQ-D *z* scores, therefore, had the same scale and direction for assessing objective and subjective cognition, respectively.

To estimate the discrepancy between objective and subjective cognition, we calculated a discrepancy score by subtracting the reversed PDQ-D *z* score from the corresponding composite NCT *z* score. The discrepancy scores would be positive or negative. In addition, it could be interpreted in a bidirectional manner. A positive discrepancy score suggested that subjective cognition was poorer than objective cognition, called cognitive ability underestimation. In contrast, a negative discrepancy score indicated that subjective cognition was superior to objective cognition, called cognitive ability overestimation.

We present each variable as percentage, mean, and standard deviation. We applied the Martinez-Iglewicz normality test to determine the distributions of continuous variables^25^. If the normality could not be rejected, we would apply parametric statistical tests to determine the statistical associations between variables and differences between groups. Otherwise, nonparametric tests would be used.

We calculated the correlations between continuous variables. For nominal data, we compared composite NCT *z* scores, PDQ-D scores, and discrepancy scores between groups. We applied the standard procedure of regression analysis to determine the independent predictors. Only the univariate variables with *p*’s≤ 0.10 were considered as eligible independent variables of composite NCT z scores, PDQ-D scores, and discrepancy scores, no subset selection approach was applied. *R* ^*2*^ and adjusted *R* ^*2*^ were used to evaluate the ratio of the sum of squares explained by a regression model.

A *p*-value of less than .05 indicated a significant prediction. All reported p-values are two-sided. The analyses were done using the NCSS 10 Statistical Software (2015) (NCSS, LLC. Kaysville, Utah, USA).

## Results

Of 57 participants, 50 (87.7%) were female. They had a mean age and years of education of 45.1 (SD = 12.8) and 11.8 (SD = 5.2) years, respectively. The PHQ-9 mean score was 13.3 (SD = 6.5). Table 1 shows other socio-demographic and clinical characteristics of the participants. Because the Martinez-Iglewicz statistic values of all continuous variables were less than 5% critical level of 1.13, the normal distributions of these data could not be rejected. Parametric tests, including Pearson’s correlation analysis, Student-t test and multiple linear regression analysis were, therefore, applied for the remainder of the data analysis.

**Table 1:** Clinical and socio-demographic characteristics of 57 patients with major depressive disorder.

Characteristics	Number
Female	50 (87.7%)
Single: Married: Divorce/Widowed	14 (24.6%): 32 (56.1%): 11 (19.3%)
First episode of MDD	33 (57.9%)
Currently taking antidepressant(s)	42 (73.3%)
Perceived disability	41 (71.93%)
Being on benzodiazepines	40 (70.2%)
	**Mean (Standard Deviation)**
Age (Years)	45.1 (12.8)
Years of education	11.8 (5.2)
PHQ-9 total score	13.3 (6.5)
CGI-S score	3.2 (0.9)
PDQ-D score	
Retrospective + Prospective subscales	12.5 (8.4)
Attention subscale	7.5 (4.8)
Planning/organization subscale	6.9 (4.3)
Total score	26.9 (15.9)
Neurocognitive test score	
Face I + Face II	65.1 (9.6)
Digit span	15.7 (3.8)
Matrix reasoning	12.4 (6.0)
Composite z score	0.0 (0.8)
Discrepancy score^b^	0.0 (1.2)

^a^≥1 SDS subscale ≥ 5 points.

^b^Composite NCT *z* score subtracted by corresponding reversed PDQ-D *z* score.

CGI-S = Clinical Global Impression, Severity Scale; PDQ-D = Perceived Deficits Questionnaire for Depression; PHQ-9 = Patient Health Questionnaire.

**Figure 1:**
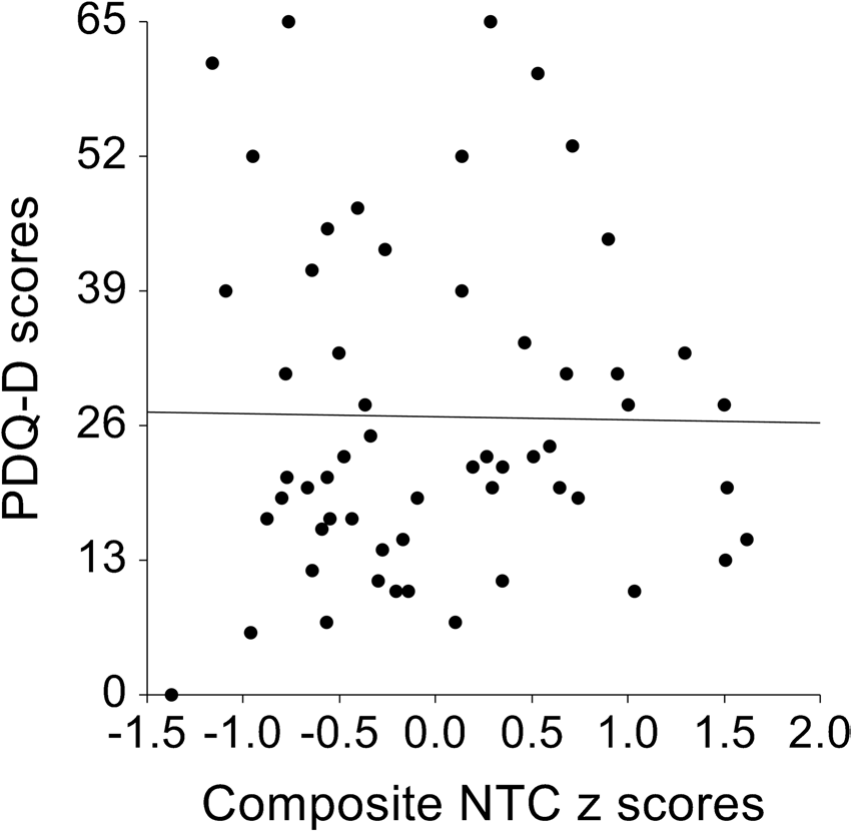
Correlation between composite *z* scores of four neurocognitive tests and perceived deficits questionnaire for depression scores (Pearson’s correlation coefficient *r* = −0.02, *p* = 0.91).

PDQ-D scores were not correlated with composite NCT *z* scores (Pearson’s correlation coefficient *r = *−0.02, *p* = 0.91) (see Fig. 1).

The univariate analysis showed that age was significantly correlated with composite NCT *z* scores (*r* = −0.49, *p* < 0.01), as was education (*r* = 0.59, *p* < 0.01, respectively). There was a trend that PHQ-9 scores were correlated with composite NCT *z* scores (*r* = 0.25, *p* = 0.07). PHQ-9 scores were significantly correlated with PDQ-D scores (*r* = 0.50, *p* < 0.01) (see Table 2). There was a trend that patients with perceived disability had a higher mean PDQ-D score than those without perceived disability (mean difference = 9, *p* = 0.05), as did currently taking antidepressants (mean difference = 9.1, p = 0.09) (see Table 3). Education was significantly associated with discrepancy scores (*r* = −0.38, *p* < 0.01), as did PHQ-9 scores (*r* = 0.55, *p* < 0.01). There was a trend that education was associated with discrepancy scores (*r* = 0.25, *p* = 0.06). In addition, there was a trend that patients with a perceived disability had a higher mean discrepancy score than those without perceived disability (mean difference = 0.7, *p* = 0.06).

**Table 2:** Socio-demographic and clinical characteristics correlated with objective cognitive function (*z* scores of 4 neurocognitive tests) and subjective cognitive function (PDQ-D score) in 57 patients with MDD.

	Pearson’s *r* for the correlation with composite NCT *z* score	Pearson’s *r* for the correlation with PDQ-D score	Pearson’s *r* for the correlation with discrepancy score^a^
Age (years)	−0.49 (*p* < 0.01)	−0.11 (*p* = 0.43)	−0.38 (*p* < 0.01)
Education (years)	0.56 (*p* < 0.01)	−0.11 (*p* = 0.41)	0.25 (*p* = 0.06)
PHQ-9 Score	0.25 (*p* = 0.07)	0.50 (*p* < 0.01)	0.55 (*p* < 0.01)

^a^Composite NCT z score subtracted by corresponding reversed PDQ-D z score. PDQ-D = Perceived Deficits Questionnaire for Depression; PHQ-9 = Patient Health Questionnaire.

**Table 3:** Differences of subjective cognitive function (PDQ-D score) and objective cognitive function (composite NCT *z* score) and between groups of major depressed patients.

	Composite NCT *z* score	Significant difference	PDQ-D score	Significant difference	Discrepancy score^b^	Significant difference
Perceived disability^a^						
No (N = 16)	−0.1 (0.8)	*t* = −0.54, *p* = 0.59	20.4 (11.0)	*t* = −1.97, *p* = 0.05	−0.5 (1.0)	*t* = −1.92, *p* = 0.06
Yes (N = 41)	0.0 (0.7)		29.4 (16.9)		0.2 (1.3)	
Gender						
Male (N = 7)	0.4 (0.8)	*t* = 1.62, *p* = 0.11	20.1 (9.5)	*t* = −1.20, *p* = 0.24	0.0 (1.2)	*t* = 0.02, *p* = 0.99
Female (N = 50)	−0.1 (0.7)		27.8 (16.5)		0.0 (1.3)	
Currently taking antidepressants						
No (N = 15)	0.2 (0.9)	*t* = 1.49, *p* = 0.14	20.9 (14.6)	*t = *−1.73, *p* = 0.09	−0.1 (1.5)	*t* = −0.47, *p* = 0.64
Yes (N = 42)	-0.1 (0.7)		29.0 (16.0)		0.0 (1.2)	
Currently taking benzodiazepines						
No (N = 17)	0.0 (0.7)	*t* = -0.11, *p* = 0.91	24.7 (17.4)	*t* = −0.67, *p* = 0.51	−0.2 (1.6)	*t* = −0.60, =0.55
Yes (N = 40)	0.0 (0.8)		27.8 (15.4)		0.1 (1.1)	

^a^≥1 SDS subscale ≥ 5 points. ^b^Composite NCT z score subtracted by corresponding reversed PDQ-D z score.

Multiple regression analysis was used to test if age, education and PHQ-9 scores significantly predicted composite NCT *z* scores. The results indicated the two predictors explained 40.0% of the variance (*R* ^*2*^ = 0.44, Adjusted *R* ^*2*^ = 0.40, *F* (3,53) = 13.68, *p* < 0.01) (see Table 4). It was found that education significantly predicted composite NCT *z* scores (*β* = 0.44, *p* < 0.01), as did age (*β* = −0.36, *p* < 0.01). All variance inflation factors were 1.13 or less suggesting no multicollinearity of the model.

**Table 4:** Summary of multiple regression analyses for variables predicting PDQ-D total and composite *z* scores (N = 57).

Variable	Composite NCT *z* score			PDQ-D score			Discrepancy score^b^		
	*b*	SE *b*	*β*	*b*	SE *b*	*β*	*b*	SE *b*	*β*
Age (years)	−0.02	0.01	−0.36**				−0.03	0.01	−0.27*
Education (years)	0.06	0.02	0.44**				0.01	0.03	0.05
PHQ-9 score	0.01	0.01	0.06	1.33	0.28	0.54**	0.09	0.02	0.48**
Perceived disability^a^				5.18	4.01	0.15	0.02	0.32	0.01
Currently taking antidepressants				13.78	3.96	0.38**			
*R* ^*2*^, Adjusted *R* ^*2*^	0.44, 0.40			0.40, 0.36			0.39, 0.34		
*F*	13.68**			11.69**			8.20**		

*p < .05, **p < 0.01. ^a^≥1 SDS subscale ≥ 5 points. ^b^Composite NCT z score subtracted by corresponding reversed PDQ-D z score.

Multiple regression analysis was used to test if PHQ-9 scores, perceived disability, and currently taking antidepressants significantly predicted PDQ-D scores. The results indicated the two predictors explained 36.0% of the variance (*R* ^2^ = 0.40, Adjusted *R* ^*2*^ = 0.36, *F* (3,53) = 11.69, *p* < 0.01) (see Table 4). It was found that PHQ-9 scores significantly predicted PDQ-D scores (*β* = 0.54, *p* < 0.01), as did currently taking antidepressants (no = 0 and yes = 1) (adjusted odd ratio = 13.78, *p* < 0.01). All variance inflation factors were 1.39 or less suggesting no multicollinearity of the model.

Multiple regression analysis was used to test if age, education, PHQ-9 scores and perceived disability significantly predicted discrepancy scores. The results indicated that the two predictors explained 34.0% of the variance (*R* ^*2*^ = 0.39, Adjusted *R* ^*2*^ = 0.34, *F* (4,52) = 8.20, *p* < 0.01) (see Table 4). It was found that age significantly predicted discrepancy scores (*β* = −0.27, *p* = 0.02), as did PHQ-9 scores (*β* = 0.48, *p* < 0.01). All variance inflation factors were 1.19 or less suggesting no multicollinearity of the model.

## Discussion

In non-elderly adults with MDD, the measures of objective and subjective cognition are not correlated. They are relatively different in many respects. Age and education are predictors of objective but not subjective cognition. In contrast, depression severity and antidepressant treatment are predictors of subjective but not objective cognition. Age and depression severity are factors predicting the discrepancy between objective and subjective cognition.

No correlation between objective and subjective cognition that was found in this study is in line with limited evidence reported earlier. A previous study reported that memory recall of depressed patients who did not report memory defects (*n* = 36) were not different from those having memory deficits (*n* = 28)^26^. In another recent study, there was a non-significant trend towards a correlation of objective and subjective cognition in 15 unipolar depressed patients (*p* = 0.06). However, such correlation disappeared after the adjustment of gender (*p* = 0.10)^12^.

The findings that age and education are associated with objective but not subjective cognition were similar to previous reports. In adult people, objective cognition is negatively correlated with age and positively associated with schooling years of education^27, 28^. The present study confirm that those correlations can be generalized from adults living in community into patients with MDD. Only a few studies have examined the correlations between age/education and subjective cognition. In the SAAD, education was not associated with both memory and concentration complaints^8^. This later study also did find the independent association between age and memory complaints. Taken together, age and education should be considered as the predictors of objective but not subjective cognition. These findings should be understandable because objective cognition is mainly based on brain function, which is superior in those with younger age and higher education.

The findings that depression severity is associated with subjective but not objective cognition were also similar to previous reports. In the SAAD, depression severity, as measured by MADRS, was independently correlated with memory and concentration complaints^8^. The present findings were also in concordance with a review summary that objective cognitive dysfunction usually persists even after symptom reduction or remission^6^. To our knowledge, no previous study reported that antidepressant treatment is a predictor of subjective cognition. In addition, this study applied a cross-sectional design and collected only few data relevant to antidepressant treatment. This later finding, therefore, should be viewed with caution and needs further confirmation.

To our knowledge, this is the first study investigating factors predicting the discrepancy between objective and subjective cognition. A mean discrepancy score of 0 suggested that, by average, this was an equally mixed sample of patients with cognitive ability underestimation and overestimation. While age was negatively correlated with discrepancy scores, depression severity was positively correlated with discrepancy score. Taken together, younger age and severe depression may predict cognitive ability underestimation, and advanced age and mild depression may also predict cognitive ability overestimation. The cognitive ability underestimation in severely depressed patients may be related with low self-esteem or pessimistic thoughts commonly found in these patients.

There were some limitations of the present study. Firstly, the sample size of 57 was small. The generally accepted ratio for a multiple linear regression model is 15 subjects for each predictor^29^. The present sample size of 57 may be too small for the regression model used to identify predictors of cognition discrepancy, which included four predictors. This sample size might not be able to detect a small to moderate effect size of difference or correlation. In addition, only seven male patients participated in this study. Generalization of the present findings to male patients with MDD may be limited. Secondly, the neurocognitive tests and the PDQ-D examined only three areas of cognition, including executive function, attention and memory. Other neurocognitive deficits commonly found in depressed patients but not included in the present study are processing (cognitive or psychomotor) speed, cognitive flexibility and verbal fluency^4, 30^. Thirdly, only few characteristics of patients were included in the predicting models of objective and subjective cognition, as well as their discrepancy. The adjusted *R* ^*2*^ of 0.40 or less suggested that the predictors can explain not more than 40% of the cases. Fourthly, despite efforts to pair the neurocognitive tests with the PDQ-D subscales, the neurocognitive tests and the PDQ-D subscales might not be perfectly matched. For example, Face I and Face II of the WMS are tests for immediate and delayed memory, but two PDQ-D subscales mainly elicit prospective and retrospective memory complaints. Fifthly, the single evaluation applied in this study might not be enough to compare the differences between objective and subjective cognition. Lastly, without the healthy controls, the present study could not determine if its findings are the nature of cognitive testing.

The present findings clearly support the discrepancy of objective and subjective cognition. Firstly, objective cognition and subjective cognition are not correlated. Secondly, predictors of subjective and objective cognition are different. The association between depression severity and subjective but not objective cognition suggests that only objective but not subjective cognition fulfil the characteristics of cold cognition.

For patients with MDD, advanced age and lower education can predict objective cognitive dysfunction. Depression can predict subjective cognitive dysfunction. More studies are needed to confirm the correlation between antidepressant treatment and subjective cognitive dysfunction. While younger age and severe depression may predict cognitive ability underestimation, advanced age and mild depression may predict cognitive ability overestimation.

## References

[1] Stedman, T. L. *Stedman’s Medical Dictionary*. (Lippincott Williams & Wilkins, 2006).

[2] Roiser JP, Sahakian BJ (2013). Hot and cold cognition in depression. CNS Spectr..

[3] Miskowiak KW, Carvalho AF (2014). ‘Hot’ cognition in major depressive disorder: a systematic review. CNS Neurol. Disord. Drug Targets.

[4] Lee RSC, Hermens DF, Porter MA, Redoblado-Hodge MA (2012). A meta-analysis of cognitive deficits in first-episode major depressive disorder. J. Affect. Disord..

[5] Iverson GL, Lam RW (2013). Rapid screening for perceived cognitive impairment in major depressive disorder. Ann. Clin. Psychiatry.

[6] Hammar A, Ardal G (2009). Cognitive functioning in major depression–a summary. Front. Hum. Neurosci.

[7] Jaeger J, Berns S, Uzelac S, Davis-Conway S (2006). Neurocognitive deficits and disability in major depressive disorder. Psychiatry Res..

[8] Srisurapanont M (2015). Subjective memory and concentration deficits in medication-free, non-elderly Asians with major depressive disorder: prevalence and their correlates. J. Affect. Disord..

[9] American Psychiatric Association. *Diagnostic and Statistical Manual of Mental Disorders*. (American Psychiatric Association, 2013).

[10] Potvin S, Charbonneau G, Juster R-P, Purdon S, Tourjman SV (2016). Self-evaluation and objective assessment of cognition in major depression and attention deficit disorder: Implications for clinical practice. Compr. Psychiatry.

[11] Ott CV (2016). Screening for cognitive dysfunction in unipolar depression: Validation and evaluation of objective and subjective tools. J. Affect. Disord..

[12] Svendsen AM, Kessing LV, Munkholm K, Vinberg M, Miskowiak KW (2012). Is there an association between subjective and objective measures of cognitive function in patients with affective disorders?. Nord. J. Psychiatry.

[13] Naismith SL, Longley WA, Scott EM, Hickie IB (2007). Disability in major depression related to self-rated and objectively-measured cognitive deficits: a preliminary study. BMC Psychiatry.

[14] Kim JM (2016). A cross-sectional study of functional disabilities and perceived cognitive dysfunction in patients with major depressive disorder in South Korea: The PERFORM-K study. Psychiatry Res..

[15] Srisurapanont M (2016). A multi-centre, multi-country, cross-sectional study to assess and describe cognitive dysfunction in Asian patients with Major Depressive Disorder (MDD). Int. J. Neuropsychopharmacol..

[16] Fehnel SE (2016). Patient-centered assessment of cognitive symptoms of depression. CNS Spectr..

[17] Sullivan MJ, Edgley K, Dehoux E (1990). A survey of multiple sclerosis: I. Perceived cognitive problems and compensatory strategy use. Can. J. Rehabil..

[18] Lam RW (2013). Psychometric validation of Perceived Deficits Questionnaire – Depression (PDQ-D) in patients with Major Depressive Disorder (MDD). Value Health.

[19] Wechsler, D. *Wechsler Memory Scale—Third Edition (WMS–III) administration and scoring manual*. (The Psychological Corporation, 1997).

[20] Wechsler, D. *Wechsler Adult Intelligence Scale - 3rd Edition (WAIS-III)*. (Harcourt Assessment, 1997).

[21] Kroenke K, Spitzer RL, Williams JB (2001). The PHQ-9: validity of a brief depression severity measure. J. Gen. Intern. Med..

[22] Guy, W. *ECDEU Assessment Manual for Psychopharmacology*. (Department of Health, Education, and Welfare, 1976).

[23] Sheehan DV, Harnett-Sheehan K, Raj BA (1996). The measurement of disability. Int. Clin. Psychopharmacol..

[24] American Psychiatric Association. *Handbook of Psychiatric Measures*. (American Psychiatric Association, 2000).

[25] Martinez J, Iglewicz B (1981). A test for departure from normality based on a biweight estimator of scale. Biometrika.

[26] Mowla A (2008). Do memory complaints represent impaired memory performance in patients with major depressive disorder?. Depress. Anxiety.

[27] de Azeredo Passos VM (2015). Education plays a greater role than age in cognitive test performance among participants of the Brazilian Longitudinal Study of Adult Health (ELSA-Brasil). BMC Neurol..

[28] Ganguli M (2010). Age and education effects and norms on a cognitive test battery from a population-based cohort: The Monongahela –Youghiogheny Healthy Aging Team (MYHAT). Aging Ment. Health.

[29] Stevens, J. *Applied multivariate statistics for the social sciences* (Lawrence Erlbaum Associates, 2002).

[30] Papakostas GI, Culpepper L (2015). Understanding and managing Cognition in the Depressed Patient. J. Clin. Psychiatry.

